# Combining Limited Multiple Environment Trials Data with Crop Modeling to Identify Widely Adaptable Rice Varieties

**DOI:** 10.1371/journal.pone.0164456

**Published:** 2016-10-10

**Authors:** Tao Li, Jauhar Ali, Manuel Marcaida, Olivyn Angeles, Neil Johann Franje, Jastin Edrian Revilleza, Emmali Manalo, Edilberto Redoña, Jianlong Xu, Zhikang Li

**Affiliations:** 1 Crop and Environmental Sciences Division, International Rice Research Institute, Los Baños, Laguna, Philippines; 2 Plant Breeding Division, International Rice Research Institute, Los Baños, Laguna Philippines; 3 Delta Research and Extension Center, Mississippi State University, Stoneville, Mississippi, United States of America; 4 Institute of Crop Science, Chinese Academy of Agricultural Sciences, Haidan District, Beijing, China; Clemson University, UNITED STATES

## Abstract

Multi-Environment Trials (MET) are conventionally used to evaluate varietal performance prior to national yield trials, but the accuracy of MET is constrained by the number of test environments. A modeling approach was innovated to evaluate varietal performance in a large number of environments using the rice model ORYZA (v3). Modeled yields representing genotype by environment interactions were used to classify the target population of environments (TPE) and analyze varietal yield and yield stability. Eight Green Super Rice (GSR) and three check varieties were evaluated across 3796 environments and 14 seasons in Southern Asia. Based on drought stress imposed on rainfed rice, environments were classified into nine TPEs. Relative to the check varieties, all GSR varieties performed well except GSR-IR1-5-S14-S2-Y2, with GSR-IR1-1-Y4-Y1, and GSR-IR1-8-S6-S3-Y2 consistently performing better in all TPEs. Varietal evaluation using ORYZA (v3) significantly corresponded to the evaluation based on actual MET data within specific sites, but not with considerably larger environments. ORYZA-based evaluation demonstrated the advantage of GSR varieties in diverse environments. This study substantiated that the modeling approach could be an effective, reliable, and advanced approach to complement MET in the assessment of varietal performance on spatial and temporal scales whenever quality soil and weather information are accessible. With available local weather and soil information, this approach can also be adopted to other rice producing domains or other crops using appropriate crop models.

## Introduction

As the staple food for half of the world population, rice is widely grown in diverse environments worldwide. Rice productivity has doubled since the Green Revolution with more than 50% of the increase attributed to continuous rice breeding efforts primarily in the irrigated system [1]. By practice, any breeding program is aimed at a specific target population of environments (TPE). It is often difficult to define the TPE for breeding efforts inclusive of rainfed areas, because rice growing environments of rainfed ecosystems vary considerably across locations and years. As a predominantly self-pollinated species, yield performances of rice varieties tend to show a significant level of genotype x environment interaction (GEI), particularly in rainfed environments. In any breeding program, it is routine to generate hundreds, or even thousands, of advanced progenies. To identify promising lines for specific TPEs from the huge numbers of advanced progenies, it requires evaluation of yield performances of large number of lines in multi-environment trials (MET), which is normally a time-consuming and very expensive process. An efficient tool that can resolve this limitation could extremely accelerate the process of varietal development.

Green Super Rice (GSR), defined as rice varieties that can produce high and stable yields using less resource input, was conceptualized and developed as the ideal rice varieties for rainfed environments. Considerable progress has been made in developing large number of GSR lines with high yield potential and tolerances/resistances to multiple abiotic and biotic stresses [2, 3, 4, 5, 6]. However, to identify promising GSR varieties suitable for specific tropical rainfed areas in Asian and African countries, there is a need to test and evaluate large number of promising GSR lines across multiple environments and to identify their TPEs. This has been a major challenge in GSR breeding efforts.

To capture GEI using a series of mathematical functions in crop models has been proven as an effective tool for evaluating yield performances of varieties in numerous environments based on key plant eco-physiological principles [7, 8]. For rice, the ORYZA2000 model has been used to predict rice growth and grain yields with high confidence level similar to other rice models in the world [9]. It has also been successfully used for performance evaluation of breeding lines or varieties over large numbers of environments [7] and environment characterization [10].

This study aims to develop and test an efficient strategy for quantifying yield performances and stabilities of GSR varieties with tolerances to multiple stresses by determining their TPEs over temporal and spatial scales using ORYZA version 3.0 (https://sites.google.com/a/irri.org/oryza2000/home).

## Materials and Methods

The modeling approach developed in this study intends to evaluate the performance of rice breeding materials across different environments. The evaluation process has four steps: 1) data collection from field experiments to determine varietal genetic coefficients to feed the crop model and from secondary data source for regional soil and weather information; 2) model calibration and validation to develop varietal genetic coefficients and evaluate the confidence level of model prediction on crop yield using experimental data; 3) conduct simulations on each rice grid cell in a given region using available soil and weather information; and 4) analysis of the simulation results to define the TPEs and evaluate varietal performance among different TPEs.

### Field experiments

The materials used in this study included eight BC_1_F_6_ GSR introgression lines with improved yields and tolerances to drought, salinity and submergence plus three check varieties (Table 1). Field experiments were conducted to test the performance of the eight GSR varieties (Table 1) during three dry seasons (2011, 2012, and 2013) and one wet season (2013) in Los Baños, Philippines (LB, 121°15’E and 14°11’N).

**Table 1:** Parentage, breeding methodology and duration of 8 green super rice (GSR) lines and check varieties used in this study.

Variety	Parentage (Cross information)	Breeding method and generation	Maturity(days after sowing) under CF*	Description
**Feng Fu Zhan (FFZ)**	Feng-Si-Zhan/Fu-Qing-Zhan 4	Pedigree breeding	115	CAAS-bred GSR variety for irrigated conditions
**GSR IR1-1-Y4-Y1**	Huang-Hua-Zhan/Yue-Xiang-Zhan//Huang-Hua-Zhan	Backcross introgression line BC1F6	120	High-yielding, irrigated cultivar with tolerance of salinity, submergence and drought
**GSR IR1-5-S8-D3-SUB1**	Huang-Hua-Zhan/OM1723//Huang-Hua-Zhan	Backcross introgression line BC1F6	120	High-yielding, irrigated cultivar with tolerance of salinity, submergence and drought
**GSR IR1-5-S10-D1-D1**	Huang-Hua-Zhan/OM1723//Huang-Hua-Zhan	Backcross introgression line BC1F6	124	High-yielding, irrigated cultivar with tolerance of salinity and drought
**GSR IR1-5-S14-Sl2-Y2**	Huang-Hua-Zhan/OM1723//Huang-Hua-Zhan	Backcross introgression line BC1F6	115	High-yielding, irrigated cultivar with tolerance of salinity and drought
**GSR IR1-8-S6-S3-Y2**	Huang-Hua-Zhan/Phalguna//Huang-Hua-Zhan	Backcross introgression line BC1F6	110	High-yielding, irrigated cultivar with tolerance of salinity, submergence and drought
**GSR IR1-8-S12-Y2-D1**	Huang-Hua-Zhan/Phalguna//Huang-Hua-Zhan	Backcross introgression line BC1F6	121	High-yielding, irrigated cultivar with tolerance of salinity and drought
**GSR IR1-12-D10-S1-D1**	Huang-Hua-Zhan/Teqing//Huang-Hua-Zhan	Backcross introgression line BC1F6	110	Aromatic, high-yielding, irrigated cultivar with tolerance of salinity and drought
**IR74371-70-1-1**	IR 55419–4*2/WAY RAREM	Pedigree breeding	110	Drought tolerant [11]
**NSICRc158**	IR 73885-1-4-3-2-1-6 (MATATAG 9)/IR70479-45-2-3//IR64680-81-2-2-1-3	Pedigree breeding	120	High Yielding under Irrigated variety [12]
**PSB Rc82**	IR 47761-27-1-3-6/PSB RC 28 (IR56381-139-2-2)	Pedigree breeding	119	High Yielding under Irrigated variety [13]

* CF = continuous flooding or under irrigated conditions

The same set of varieties was also tested in Nueva Ecija, Philippines (NE, 120°56’E and 15°42’N) during the 2013 dry and wet seasons (Table 2). The experiments conducted in 2011 and 2012 had two types of water management: 1) continuously flooded throughout the season; and 2) switching from continuously flooded to rainfed condition after panicle initiation. All experiments in 2013 were under fully irrigated conditions with standard practices of nitrogen fertilizer application and other crop management.

**Table 2:** Experiments and associated varieties implemented in Los Baños (LB) and Nueva Ecija (NE), Philippines, during the dry seasons of 2011 (2011-DS), 2012 (2012-DS), 2013 (2013-DS) and wet season of 2013 (2013-WS). RF indicates switching from continuously flooded to rainfed condition after panicle initiation while CF indicates continuously flooded throughout the season. The datasets were marked for calibration (C) and evaluation (E).

Variety	Experiments					
	2011-DS-RF	2011-DS-CF	2012-DS-RF	2012-DS-CF	2013-DS-CF	2013-WS-CF
**FFZ**	C	C	E	E		
**GSR IR1-1-Y4-Y1**			C	E		
**GSR IR1-5-S8-D3-SUB1**			C	E		
**GSR IR1-5-S10-D1-D1**	E	C	C	E		
**GSR IR1-5-S14-Sl2-Y2**			C	E		
**GSR IR1-8-S6-S3-Y2**			C	E		
**GSR IR1-8-S12-Y2-D1**	E	C	C	E		
**GSR IR1-12-D10-S1-D1**	E	C	C	E		
**IR-74371-70-1-1**	E	C	C	E		
**NSICRc158**	C	C			C(LB), E(NE)	E(LB, NE)
**PSBRc82**	E	C	C	E	C(LB), E(NE)	E(LB, NE)

Phenology development was recorded and destructive samples were collected from all field experiments to determine the leaf area index (LAI) and biomass weights for green leaves, dead leaves, stems, and panicles. Grain yields were determined in two 3 m^2^ areas at physiological maturity. Soil water potential at soil depth of 15 cm was measured in rainfed fields using MPS 2 sensors (Decagon Devices Inc., Pullman WA) connected to a Decagon EM50 data logger.

The measured data under different crop management strategies and climate-soil conditions were used to parameterize the genetic parameters of the tested GSR lines and checks to evaluate the reliability of ORYZA (v3) in representing the growth, development, and yields of the tested rice varieties across different environments.

### Modeling study

#### General description of ORYZA version 3.0

Evaluations conducted under various environments and crop management practices have established the reliability of ORYZA2000 in predicting rice growth, development, and yield [14]. This model has been successfully used in evaluating the performance of breeding lines in various environments [7] under different water and nitrogen management systems [5, 10, 15, 16, 17]. The ORYZA (v3) is an improved version of ORYZA2000 with additional functions to quantify the effects of drought, nitrogen deficiency, and irrigation management [18] (https://sites.google.com/a/irri.org/oryza2000/home). Unlike ORYZA2000 [19], most of the variety parameters used by ORYZA (v3) are genetic parameters, and the effects of environment on photosynthesis, assimilate partitioning, growth of crop organs, leaf area development, and water and nitrogen uptake have been integrated into the model with extra parameters representing responses of specific varieties.

#### Model calibration for varietal parameterization

The observed data from experiments marked by “C” (Table 2) was used for variety parameterization in the process of model calibration. The experiment-specific crop management practice and weather information were used correspondingly in each experiment. Under rainfed conditions, soil water potential was used as input for variety parameterization. The parameters controlling and affecting leaf development, light interception, assimilate partitioning, leaf death, drought tolerance, rooting depth, and nitrogen uptake were calibrated using an auto-calibration tool to minimize the differences between the measured and simulated values on LAI, total above-ground biomass (AGB), and biomass of green leaves, dead leaves, stems, and panicles.

The auto-calibration program was developed based on differential evolution algorithms of global optimization [20] and applied on the earlier studies [21, 22] for parameterization of ORYZA2000. The documentation of varietal parameterization using the auto-calibration program is available online (https://sites.google.com/a/irri.org/oryza2000/downloads/ new-release/download-new-version). The varietal parameters used in this study were fixed for ensuring that the differences between simulated and measured values are similar to the coefficients of variation (CV) of field measurements [23, 24], the desired target differences were set to 5% of the measured values for storage organ biomass, 10% for green leaf and total AGB, and 15% for LAI and dead leaf biomass. The initial values of varietal parameters were from a popular variety, IR72, and their variation ranges were assumed to be 25 to 50% to ensure that the calibrated parameter values are physiologically reasonable. The values of key varietal parameters for each GSR variety developed from the calibration process were presented in S1 Table (Hereafter, ‘S’ after table/figure numbers indicates supporting information).

#### Validation of model prediction

Model predictions on rice growth and yield must be validated using independent experiments before the model can be used for further simulations over different environments. To ensure the predictability of ORYZA (v3) for the growth and development of different varieties under different environments, the model was initially evaluated against a standard set of measurement data from actual field experiments as indicated by ‘E’ in Table 2.

The experiment-specific weather and soil data, as well as crop management information, were fed to the model for validation. Measurements on the actual and simulated sequential AGB, panicle biomass (PB), and commercial grain yield (GY) from the experiments for all GSR and check varieties were integrated into X and Y data pairs for each sampling date. With the data pairs, the statistical analyses were conducted to quantify the differences between measured actual (*X*) and simulated (*Y*) values for AGB, PB, and GY. The calculated statistical parameters were the linear regression parameters for slope (*α*), intercept (*β*), and correlation coefficient (*R*^*2*^), and the Student’s t-test with unequal means assumption (*P(t)*). In addition, the normalized root mean square errors (*RMSE*_*n*_) and index of modeling agreement (*M*_*eff*_) [25] were calculated using Eqs 1 and 2, where *n* is the number of measurements, *i* is the data pair index, and $\bar{X}$ is the average of all observations for a variable.

$$RMSE_{n}=\frac{{\left(n\sum_{i=1}^{n}{\left(Y_{i}-X_{i}\right)}^{2}\right)}^{0.5}}{\sum_{i=1}^{n}X_{i}}\times 100$$ (1)

$$M_{eff}=1-\frac{\sum_{i=1}^{n}{\left(Y_{i}-X_{i}\right)}^{2}}{\sum_{i=1}^{n}{\left(|Y_{i}-\bar{X}\left|+\right|X_{i}-\bar{X}|\right)}^{2}}$$ (2)

Model reproduces experimental data best when *α*, *R*^*2*^ and *M*_*eff*_ are close to 1, *β* is close to 0, *P(t)* is larger than 0.05, and *RMSE*_*n*_ is similar to the *CV* of measured values.

#### Simulations with numerous environments

GEI needs to be quantified using thousands of simulations in various environments in order to achieve the study targets. Consequently, the simulations were designed to have the same crop management across environments for all varieties to ensure that the variation in rice growth and development will come from the GEI. In this study, crop management was excluded as a factor of environment because it is an integration of human activities and could be modified easily.

All GSR varieties used in this study were bred for drought-prone environments, thus, the simulations were designed for rainfed conditions. The current rice cultivation regions in Southern Asia were gridded at 15 arc-minute geo-resolution, with each grid cell assigned as one environment, thereby producing 3796 environments. The corresponding historical weather (across 15 years) and soil information of each grid cell was extracted from IRRI database and from the World Inventory of Soil Emission Potential (WISE), respectively [26], to determine the temporal and spatial variations of the target environments. Temporally, the 15 years of weather information from 1998 to 2012 explained the dynamics and variation of the environment season by season. Spatially, the environments of adjacent grid cells may be similar to or significantly different from each other due to varying weather and soil information.

For each GSR variety and each environment, two groups of simulations were designed to differentiate the performance of the crop under the two types of water management practices—continuously flooded (group 1) and rainfed (group 2) in Table 2. For both groups, fertilizer was fully supplied, and rice was assumed to grow under conditions without any biotic-stress. Therefore, radiation and temperature were the only limiting factors for rice growth in group 1, while soil moisture was an additional limiting factor in group 2. In each group, 24 sowing dates per year were included, starting from 1 January at 15-day intervals, over 14 years (from 1998 to 2011). The rice crop sown in the later seasons of 2011 grew through early 2012 and required the weather information for 2012 for simulation. In total, 672 simulations (24 sowing dates y^-1^ × 14 years × 2 groups) were implemented for each variety in each environment. Other crop management practices, except for water management, were completely the same for both groups. The field bund height was set to 10 cm and the seedlings were transplanted at 14 days after sowing with a density of 66 seedling m^-2^.

The model predicted commercial GY (grain biomass with 14% moisture). The average drought index (varying from 0 to 1 for severe to no drought stresses) in the vegetative and reproductive stages was used for varietal performance evaluation over a large number of environments and for the identification of TPEs.

### Analysis of data

#### Simulation data cleaning

Three variables were extracted from the outputs of simulations for each variety in each environment and each group, namely GY, average daily drought stress index in the vegetative stage (*DIV*) and reproductive stage (*DIR*). The outputs of each variable in groups 1 and 2 (i.e. irrigated and rainfed water management, respectively) were organized into matrices *P* and *A*, respectively (Eqs 3 and 4), where *p* and *a* stand for the simulated values of a variable among *GY*, *DIV*, and *DIR* from groups 1 and 2. The subscripts *i* and *j* represent the indices of sowing dates in a year and years of simulation, respectively. Subscript *i* changes from 1 (first sowing date on 1 January) to *n* (last sowing date), while *j* varied from 1 (start year 1989) to *m* (last year of simulation). In this study, the *n* and *m* were 24 and 14, respectively.

$$P=\left|p_{ij}\right|\;\;\left(i=1,2,\cdots ,n;j=1,2,\cdots ,m\right)$$ (3)

$$A=\left|a_{ij}\right|\;\;\left(i=1,2,\cdots ,n;j=1,2,\cdots ,m\right)$$ (4)

The matrix *P* was only organized for *GY*, assigned as *PY*, but *A* was organized for variables *GY*, *DIV*, and *DIR*, indicated as *AY*, *AV*, and *AR*, respectively. In *PY*, any zero value of *p*_*ij*_ implied that the *i*^*th*^ sowing date was not suitable for rice growth. Therefore, the corresponding *p*_*i*._ and *a*_*i*_ in all four matrices were removed and excluded in the succeeding analyses.

#### Classification of TPE

To define the characteristics of each environment, the matrices *PY*, *AY*, *AV*, and *AR* of all varieties were combined into four big matrices of *PY*′, *AY*′, *AV*′, and *AR*′, respectively. The TPE for rainfed rice was classified based on information on the best rainfed season in each environment.

To identify the best rainfed season, the average rainfed yield (*RY*) and *CV* over the years and all varieties under rainfed condition were calculated for each sowing date from *AY*′ (Eqs 5 and 6), where *c* is the number of varieties in this study, subscript *v* denotes varieties, and *i* stands for sowing seasons. The 24 sowing dates were tested to derive *RY* and a maximum of 24 values were derived for *CV*. The best rainfed season was selected from the top three *RY* for the smallest *CV*.

$$RY_{i}=\frac{\sum_{v=1}^{c}\sum_{j=1}^{m}a_{ijv}}{m\times c}$$ (5)

$$CV_{i}=\frac{\sqrt{\frac{1}{m\times c}\sum_{v=1}^{c}\sum_{j=1}^{m}{\left(a_{ijv}-a_{i}^{\prime }\right)}^{2}}}{AY_{i}}$$ (6)

For each environment, the data of the best rainfed season was extracted from *PY*′, *AY*′, *AV*′, and *AR*′ to data arrays *py*_*jv*_, *ay*_*jv*_, *av*_*jv*_, and *ar*_*jv*_ for irrigated and rainfed grain yields, and the drought indices at the vegetative and reproductive stages, in which *j* and *v* are the year and variety indices among all tested varieties and years. These four arrays were used to determine the TPE class of a given environment.

For each environment, the TPE class for rainfed rice was defined by the severity and type of drought stress it was subjected to. The severity of drought stress was presented by the frequency (*f*_*75*_) at which *ay*_*jv*_*/py*_*jv*_ is lower than 0.75. Here, we assumed that the yield reduction by 25% caused by drought is acceptable for a single season, but a higher frequency of such reduction among seasons was considered a significant drought impact. Severity of drought stress was classified into three groups: 1) severe drought stress for f_75_≥50%; 2) moderate drought stress for 25%≤f_75_<50%; and 3) none to mild drought stress for f_75_<25% (Table 3).

**Table 3:** TPE classification of environment in the study area based on the drought severity and drought timing. The *f*_*75*_ is simply the frequency of the rainfed yields occurring less than 75% of irrigated yield.

TPE	Drought Timing	Drought Severity
**L1**	**Vegetative**	**None or Mild Drought (*f***_***75***_**<25%)**
**L2**	**Reproductive**	
**L3**	**Vegetative + Reproductive**	
**M1**	**Vegetative**	**Moderate Drought (25%≤*f***_***75***_ **<50%)**
**M2**	**Reproductive**	
**M3**	**Vegetative + Reproductive**	
**S1**	**Vegetative**	**Severe Drought (*f***_***75***_**≥50%)**
**S2**	**Reproductive**	
**S3**	**Vegetative + Reproductive**	

The impact of drought stress is highly related to the timing of its occurrence in the different rice growth stages. Drought stress is relatively more harmful when it occurs in the reproductive rather than the vegetative stage [27]. In this study, drought stress was classified into three types according to the growth stage at which it occurred: 1) vegetative; 2) reproductive; and 3) mixed, when drought stress occurred in both vegetative and reproductive growth stages.

To determine the drought type of an environment, $\bar{av}$ and $\bar{ar}$ were derived from *av*_*jv*_ and *ar*_*jv*_ as average values of all their elements. Drought was vegetative type if $\bar{av}$ is smaller than $\bar{ar}$, or reproductive type if $\bar{ar}$ is smaller than 90% of $\bar{av}$, otherwise, the drought was mixed type (Table 3). Finally, all environments were classified into 9 TPE groups based on the GEI generated by simulation results of ORYZA (v3).

#### Yield stability and adaptability of variety

Yield stability of a given variety across environments was evaluated with simulated grain yields under irrigated and rainfed conditions among the best rainfed rice seasons. For a variety, the irrigated and rainfed yields in the best rainfed season were extracted from each environment to form two new data matrices YP and YA (Eqs 7 and 8), where *j* stands for the simulation years changing from 1 to *m* (Eqs 1 and 2), *k* stands for the number of environments, and *q* is the total number of environments.

$$YP=\left|yp_{jk}\right|\;\left(j=1,2,\cdots ,m;k=1,2,\cdots ,q\right)$$ (7)

$$YA=\left|ya_{jk}\right|\;\left(j=1,2,\cdots ,m;k=1,2,\cdots ,q\right)$$ (8)

Five indicators, *γ*, *δ*, *λ*, *φ* and *ψ*, were derived from the data of YP and YA to quantify the yield stabilities of a variety in a class of TPE or all tested environments where parameter *γ* is the average rainfed yield derived from *YA*; *δ* presents how far the rainfed rice yield is from the irrigated rice yield (Eq 9); λ is the coefficient of variation of rainfed yield in *YA* (Eq 10), representing the yield variation cross over both spatial and temporal scales; *φ* is the spatial variability of yields among environments; and *ψ* is the temporal variability of yield among different growth seasons (Eqs 11 and 12). The parameters *λ*, *φ*, and *ψ*, represent the yield stability over all environments, or on a given TPE, depending on the yield dataset used in the analysis. Variety has higher yield stability when the values of these parameters are lower.

$$\delta =\frac{\sqrt{m\times q\times (\sum_{j=1}^{m}\sum_{k=1}^{q}{\left(yp_{jk}-ya_{jk}\right)}^{2})}}{\sum_{j=1}^{m}\sum_{k=1}^{q}yp_{jk}}$$ (9)

$$\lambda =\frac{\sqrt{\frac{1}{m\times q}\sum_{j=1}^{m}\sum_{k=1}^{q}{\left(ay_{jk}-\gamma \right)}^{2}}}{\gamma }$$ (10)

$$\varphi =\frac{\sqrt{\frac{1}{q}\sum_{k=1}^{q}{\left(\left(\frac{1}{m}\sum_{j=1}^{m}ay_{jk}\right)-\gamma \right)}^{2}}}{\gamma }$$ (11)

$$\psi =\frac{\sqrt{\frac{1}{m}\sum_{j=1}^{m}{\left(\left(\frac{1}{q}\sum_{k=1}^{q}ay_{jk}\right)-\gamma \right)}^{2}}}{\gamma }$$ (12)

The analyses above were implemented for all environments and 9 TPE classes. To derive the five indicators for all environments as an entity, the data in PY and AY were used. For each TPE class in Table 3, the five indicators were derived from the YP and YA, which were categorized into 9 groups based on TPE classes (Table 4).

**Table 4:** The datasets used to determine TPE Classes and yield stability in the TPE. YP and YA are the irrigated and rainfed rice grain yields in the best rainfed season. The λ is the coefficient of variation of rainfed yield in YA, φ is the spatial variability of yields among environments, and the ψ is the temporal variability of yield among different growth seasons.

TPE Classes	Yield datasets to define TPE	Yield stability (= 1.0—value of the parameter)
**L1**	YP_l1_ and YA_l1_	λ, φ and ψ for domain of TPE: L1
**L2**	YP_l2_ and YA_l2_	λ, φ and ψ for domain of TPE: L2
**L3**	YP_l2_ and YA_l_	λ, φ and ψ for domain of TPE: L3
**M1**	YP_m1_ and YA_m1_	λ, φ and ψ for domain of TPE: M1
**M2**	YP_m2_ and YA_m2_	λ, φ and ψ for domain of TPE: M2
**M3**	YP_m3_ and YA_m3_	λ, φ and ψ for domain of TPE: M3
**S1**	YP_s1_ and YA_s1_	λ, φ and ψ for domain of TPE: S1
**S2**	YP_s2_ and YA_s2_	λ, φ and ψ for domain of TPE: S2
**S3**	YP_s3_ and YA_s3_	λ, φ and ψ for domain of TPE: S3

A better variety should have a large value of *γ* to indicate high productivity in drought-prone environments, and a small value of *δ* to indicate strong tolerance to drought stress or low risk in drought-prone environments. A small value of *λ* is attributed to good yield stability in spatial and temporal dimensions, or even a good spatial or temporal yield stability with the small value of *φ* or *ψ*.

Moreover, the values of *λ*, *φ* and *ψ* derived from the whole matrices of PY and AY also represented the properties for varietal adaptability among TPEs which may vary from severe to no drought stress conditions. The good adaptability for many different kinds of TPEs does not necessarily mean a good productivity and lower risk to drought stress. For a rice breeder, a variety with low values of *δ*, *λ*, *φ* and *ψ*, and a high value of *γ* over different kinds of environments is a good set of selection criteria. The selection would be very difficult for all TPE classes but is achievable for a few TPE classes. For a local farmer, a variety with lower values of *λ* and *ψ* and a higher value of *γ* is desirable, while a low value of *δ* implies easy crop management in rainfed rice production systems.

#### The cluster analysis

Hierarchical clustering analysis was conducted to directly show the difference between the GSR and the check varieties in terms of plant physiological parameters using the ORYZA (v3) model and varietal adaptability. The varietal performance in a given TPE or across all TPEs was clustered into five groups based on the five parameters *γ*, *δ*, *λ*, *φ* and *ψ* to rank the performances of the varieties from 1 (best) to 5 (worst).

## Results

### The reliability of ORYZA (v3) for production prediction

Following the calibration and validation processes, each variety used in this study was parameterized and evaluated in independent experiments (Table 2). In comparing the simulated to measured values, ORYZA (v3) performed well in representing the three key plant growth variables–AGB, PB, and GY–in both calibration and validation datasets for all varieties (Table 5). The estimations on AGB, PB, and GY were reliable for individual varieties because all statistical indicators were close to the desirable values (Table 5), despite different values among varieties (S2 and S3 Tables). The graphical analysis shows that more than 90% of the simulated plant biomass and GY values were within the range of confidence defined by CV of measurements (S1 Fig). Particularly, the similar accuracies in estimating biomass accumulation and grain yields in rainfed conditions as those in full irrigated conditions implied that drought stress in rainfed was a reliable estimate.

**Table 5:** Statistical analysis for the calibration and validation datasets of all tested varieties in this study. AGB is above-ground biomass, PB is panicle biomass, and GY is grain yield.

Variable	Data pairs	*R*^*2*^	*α*	*β*	*p(t)*	*RMSEn (%)*	*M*_*eff*_
	Calibration dataset						
AGB	102	0.969	284.959	0.919	0.470	8.592	0.998
PB	50	0.973	120.395	0.955	0.492	4.566	0.999
GY	26	0.898	567.020	0.849	0.493	8.987	0.993
	**Validation dataset**						
AGB	116	0.955	191.209	0.929	0.425	7.851	0.998
PB	55	0.971	-93.475	0.971	0.357	6.636	0.999
GY	27	0.885	314.758	0.871	0.305	9.282	0.990

In summary, ORYZA (v3) was able to represent the dynamics of AGB and PB during the growing season, and also well predicted the end-season GY for all tested varieties and environments. The simulated values on yield, biomass accumulation, and drought stress were reliable inputs for the classification of TPE and evaluation of varietal performance in a large number of different environments.

### Classification of TPE based on GEI

**Fig 1:**
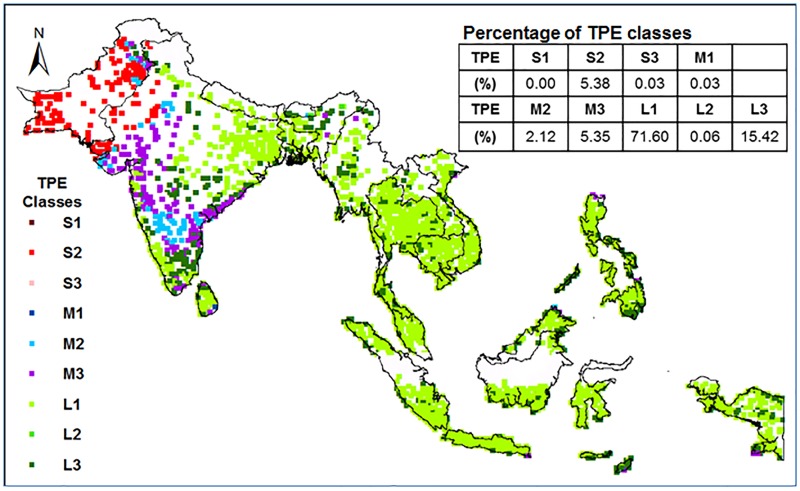
The TPE classes of the tested environments in South Asia. The TPE classes of drought stress to rainfed rice at possible areas and the best rainfed season.

Using the calibrated and validated crop parameters, ORYZA (v3) predicted the yields of all varieties based on GEI (Table 1) in 3796 environments (i.e. soil and climate combinations) in Southern Asia. Using the predicted yields under fully irrigated and rainfed conditions, these environments were classified into 9 TPE classes depending on drought severities and time of drought occurrence relative to the crop growth stage in the best local rainfed season (Fig 1). The major TPE was identified with mild drought stress at the vegetative stage, with 15.4% of the total environments under mild drought throughout the growth season. Moderate drought stress was mainly affecting the whole growth season or reproductive stage while severe drought mainly occurred in the reproductive stage for 5.4% of the environments. There was no severe drought stress for the best rainfed season in Southeastern Asia. In this region, there was no significant yield penalty for rainfed rice if it is grown in the best local rainfed season. Rainfed rice would suffer severe drought stress in western parts of South Asia, which implies that rice yield could not reach 75% of the irrigated rice yield in more than 50% of the best rainfed seasons.

### Yield stability of GSR varieties

**Fig 2:**
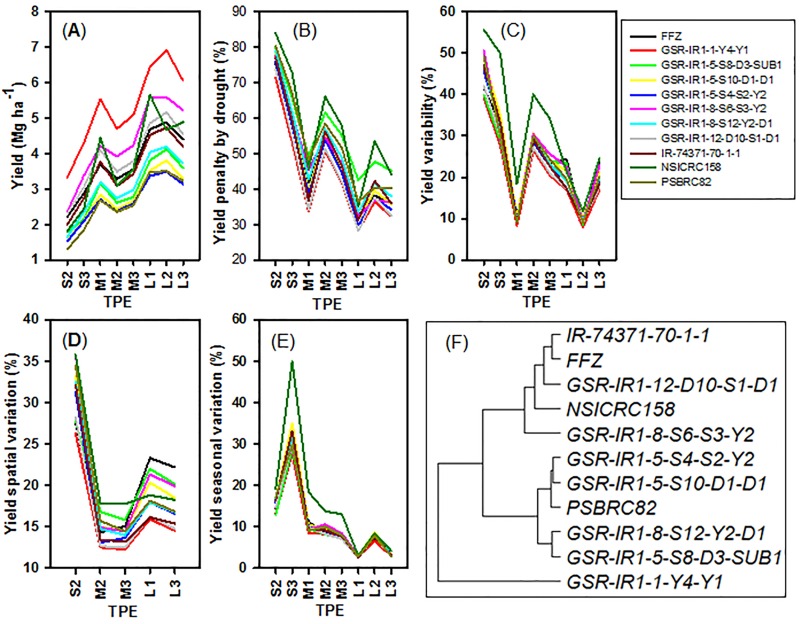
The rainfed rice yields, yield variation and stability of tested varieties over different TPEs. Yield and yield spatial variation.

The TPE type for severe drought stress in the vegetative stage (S1) did not exist for the best rainfed rice season in Southern Asia (Fig 1). Consequently, S1 was excluded in all succeeding analyses (Fig 2). The TPE types, S3, M1, and L2 only occurred in a few environments, hence, were also excluded in the next analysis for yield spatial variation (Fig 2D).

It was not surprising that the rainfed rice yields increased for all varieties as the drought stress changed from severe to mild stress among environments (Fig 2A), but the yield penalty caused by drought and the yield under severe drought stress were significantly different (Fig 2A and 2B). The drought stress at the reproductive stage had a stronger impact on rainfed rice production than the other two types of drought stresses (vegetative only, and combined vegetative and reproductive drought stresses) (Fig 2B). Yield variability followed a similar trend of yield penalty among TPEs (Fig 2C). Similar to yield penalty and variation (Fig 2B and 2C), the seasonal variation of yield also decreased as drought stress decreased (Fig 2E). However, this does not hold true for yield spatial variation (Fig 2D), where yield spatial variation was higher in mild drought stress environments than in moderate drought stress environments. As expected, the yield spatial and seasonal variations were much higher in severe drought stress than in moderate and mild drought stress environments because the impact was normally magnified in severe drought stress environments. This implies that the little changes on drought severity and/or occurring time would result in large changes in rice production.

Considering the yields and their stability over different TPEs for 3796 environments, the variety GSR-IR1-1-Y4-Y1 was outstanding among all other varieties due to its significantly higher GY, relatively lower yield penalty of drought, and relatively good grain yield stability over locations and seasons (Fig 2C–2E). Varying crop characteristics resulted in the different performance of all other varieties (Fig 2F). GSR-IR1-8-S6-S3-Y2 was also demonstrated to perform better than the check varieties PSBRC82, NSICRc158, and IR74371-70-1-1 and was found to have good adaptability to all environments. The varieties GSR-IR1-5-S8-D3-SUB1 and GSR-IR1-8-S12-Y2-D1 were similar to the check varieties PSBRC82 and NSICRc158 (Table 6). In severe drought environments, where the yield penalty of drought may reach 85%, only GSR-IR1-1-Y4-Y1 and GSR-IR1-8-S6-S3-Y2 had better adaptability than or equal to the check variety PSBRc82 (Table 6). GSR-IR1-1-Y4-Y1, GSR-IR1-5-S8-D3-SUB1, GSR-IR1-5-S10-D1-D1, GSR-IR1-8-S6-S3-Y2, and GSR-IR1-8-S12-Y2-D1 were relatively more competitive with the check varieties under moderate and mild drought stress environments. Except for GSR-IR1-1-Y4-Y1 and GSR-IR1-5-S10-D1-D1, the other GSR varieties did not have an advantage to adapt to drought stresses at reproductive or combined vegetative and reproductive stages, while most GSR varieties adapted well to drought stress at the vegetative stage.

**Table 6:** Adaptability ranking of the varieties to different levels of drought stress. Severity can be severe (S), moderate (M), and none to mild (L) which can occur at vegetative (V), reproductive (R), and combined vegetative and reproductive (V+R) timing.

VARIETY	Drought severity			Drought timing			Sum
	S	M	L	V	R	V+R	
FFZ	4	5	4	5	3	5	5
GSR-IR1-1-Y4-Y1	1	1	1	1	1	1	1
GSR-IR1-5-S8-D3-SUB1	5	3	2	2	3	2	3
GSR-IR1-5-S10-D1-D1	5	3	2	2	2	3	4
GSR-IR1-5-S14-S2-Y2	5	4	3	3	3	3	4
GSR-IR1-8-S6-S3-Y2	2	2	3	4	2	2	2
GSR-IR1-8-S12-Y2-D1	5	3	2	2	3	2	3
GSR-IR1-12-D10-S1-D1	4	4	3	4	3	4	4
IR-74371-70-1-1	3	5	4	5	3	5	5
NSIC Rc158	3	3	3	4	3	3	3
PSBRc82	2	4	3	3	3	2	3

### Identification of potential adaptation regions for outstanding varieties

**Fig 3:**
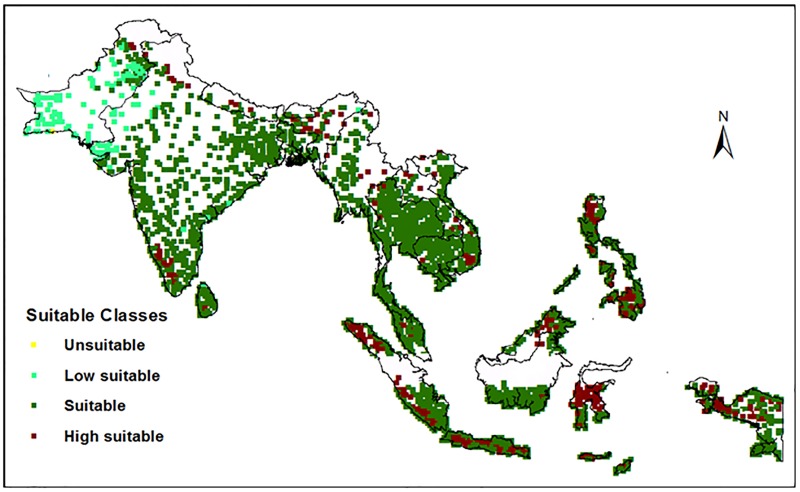
The potential dissemination areas of GSR-IR1-1-Y4-Y1. The potential dissemination regions of the identified outstanding variety GSR-IR1-1-Y4-Y1 in Southern Asia.

The performance of GSR-IR1-1-Y4-Y1 was outstanding among all other GSR and check varieties in all types of TPEs, hence, having a high potential for wide dissemination in Southern Asia. The suitability of this variety in different regions in Southern Asia should be explored (Fig 3), with a high suitability found in Southeast Asia and a low suitability in some western parts of South Asia (i.e. Pakistan and West India).

## Discussion

### The consistency between field experiment and simulation

ORYZA (v3) well represented the biomass accumulation and final GY at site-specific conditions (Table 5, S2 and S3 Tables, and S1 Fig). The difference in rice yield was not statistically significant between the model predictions and the actual observations in the field experiments, which confirmed that ORYZA (v3) is able to provide a reliable estimation of rice yield in various environments.

Using these reliable rice yield predictions, the performance of GSR varieties was evaluated across a large number of environments in comparison with check varieties. The same evaluation was also conducted using data from field experiments under limiting environments. The evaluation results from the simulated data were inconsistent with the results from the measured GYs (Table 7). The key parameters were not comparable between the results from the field experiments in limited environments and the simulation in a large number of environments in terms of average yield, variation, and penalty of yield in drought-prone environments, resulting in a different performance ranking of the tested varieties (Table 7, *MET*_*order*_ vs. *P*_*order*_). This result suggests that the sampled environments of the field experiments did not cover the highly diverse environments across the region in simulation. While the varietal performance ranking was constrained only for experimental sites and 14 best rainfed seasons (S4 Table), the *MET*_*order*_ and *S*_*order*_ still did not completely agree with each other, but the ranking difference between them was insignificant (Table 7). However, *S*_*order*_ of the top performing varieties corresponds significantly to *MET*_*order*_ (R^2^ = 0.86, p<0.0001) in this case. In other words, with site specific soil and weather information, the ORYZA (V3) model was able to correctly evaluate the varietal performances in the MET experiments for specific sites. However, the accuracy in evaluating varietal performances using the model approach was still constrained by the quality of input information on weather and soil. Further study should be conducted to assess how these kinds of information cause inconsistency of varietal performances, particularly under extreme (good and bad) environments. In practice, any variety should be definitely considered promising, provided it had a top ranking in grain yield in both field experiments under a limited number of environments and simulation results from a larger number of environments. In this study, the GSR IR1-1-Y4-Y1-Y1 and GSR IR1-8-S6-S3-Y2 were confirmed to be the most promising varieties for the rainfed areas using this approach, as they showed 10–20% and 20–40% grain yield advantages over the advanced check NSICRC158 and drought tolerant check IR74371-70-1-1, respectively, across large number of environments.

**Table 7:** Results derived from field experiments in limited environments and simulations under a large number of environments.

ID	Field experiment						Large simulation				
	N	Yield	CV	Yield penalty	MET_order_	S_order_	N	Yield	CV	Yield penalty	P_order_
FFZ	2x2	4.16	0.83	81.8	5	**5**	3796x14	4.42	0.29	39.0	5
GSR IR1-12-D10-S1-D1	2x3	3.84	0.84	82.0	8	**6**	3796x14	4.58	0.23	35.6	4
GSR IR1-1-Y4-Y1-Y1	2x1	4.23	0.45	61.9	4	**3**	3796x14	6.11	0.22	35.3	1
GSR IR1-5-S10-D1-D1	2x2	4.12	0.66	70.9	6	**8**	3796x14	3.31	0.27	39.1	9
GSR IR1-5-S14-S2-Y2	2x1	5.07	0.47	64.3	2	**2**	3796x14	3.17	0.25	37.4	11
GSR IR1-5-S8-D3-Sub1	2x1	6.29	0.59	74.5	1	**1**	3796x14	3.58	0.28	47.9	8
GSR IR1-8-S12-Y2-D1	2x2	3.16	0.91	86.8	11	**9**	3796x14	3.78	0.26	40.6	7
GSR IR1-8-S6-S3-Y2	2x1	4.68	0.49	66.0	3	**4**	3796x14	5.25	0.28	39.2	2
IR74371-70-1-1	2x2	3.87	0.65	71.4	7	**7**	3796x14	4.25	0.24	38.8	6
NSIC Rc158	3x1	3.67	0.38	91.6	10	**11**	3796x14	5.16	0.30	43.9	3
PSBRc82	2x1+3x1	3.73	0.68	69.0	9	**10**	3796x14	3.26	0.26	42.6	10

MET_order_ is the varietal performance rank of all 11 varieties from the best (1) to the worst (11) ranked by the data from MET field experiment, S_order_ is the varietal performance rank using simulation data under MET site-specific condition, and P_order_ is the varietal performance rank using simulation data under numerous environments. The N is number of environments while CV presents the coefficient of variation of yields among environments.

### Evaluation from GEI to TPE

**Fig 4:**
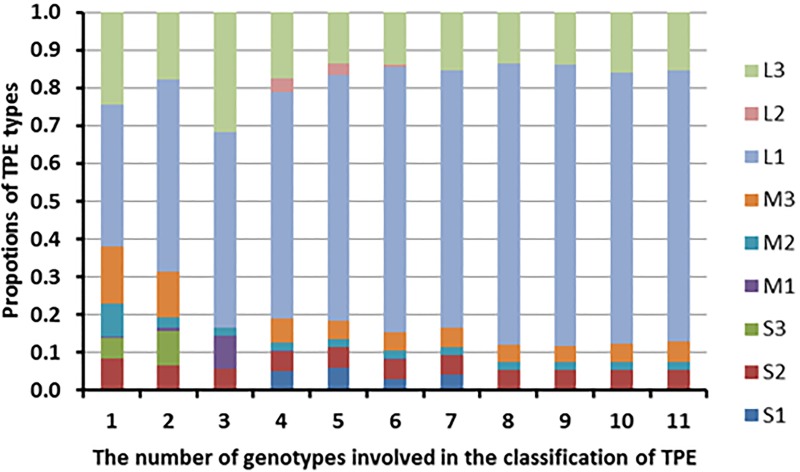
Proportional change in TPE types with increase in number of genotypes used for TPE classification. The TPE types in this study were defined by the drought stress severity and types.

In the ORYZA (V3) model, the genotype is characterized by a series of quantitative parameters, including the weather, soil, and crop management information. These parameters are inputs for simulations, in which the genetic parameters for a rice variety are constant and the environment parameters constantly change. As a result, GEI, which was simply quantified by the simulated varietal yields through a series of eco-physiological processes in the model, may vary considerably due mainly to various environments. Consequently, environments can be classified using the GEI results represented by the simulation yields in this case. Similar approach has been proven as an effective and widely used method to classify environments of crop growth [28, 29, 30, 31]. It was used as classification of TPE for rice growth [32, 33]. In this study, the quantification of GEI by the ORYZA (V3) model used a set of variables for production environments as well as the number of environments and seasons [34], for classifying the TPEs of the rainfed ecosystems. However, the number and types of genotypes could significantly affect the classification of TPE (Fig 4). Thus, the number of different genotypes to be used in the model should not be less than ten in order to accurately define the types of TPE from GEI.

It was evident that the variation of genotypes also affected the confidence levels pertaining to the definition of TPE types. In this study, all genotypes were assumed to have some degree of drought tolerance. The proportion of TPE with severe drought would increase when more drought-sensitive genotypes were used. Moreover, the best rainfed seasons used in the classification of TPE may differ to local rice farming seasons, introducing bias in the classification, which should be addressed in future studies. On the other hand, the defined TPE, as in severe drought, would be very useful in the selection of drought tolerant varieties. The drought tolerance of a new line would be better than the varieties used in this study if it performed better in a typical drought TPE.

## Conclusions

ORYZA (v3) was able to reliably predict the total above-ground and panicle biomass, as well as the final grain yield of rice. The uncertainty of around 10% on grain yields was comparable to the coefficient of variation of the field measurements on these variables. In this study, calibration and validation were carried out in multiple environments. The reliable predictions on grain yield ensure the capacity of ORYZA (v3) to evaluate the performances of these calibrated varieties in a large number of environments. The crop modeling approach was demonstrated to effectively evaluate varietal performances over large numbers of environments with minimal costs and time. High quality evaluation on varietal performance using the modeling approach can be achieved with improved soil and weather information. The developed approach can also be used for other rice growing regions whenever the crop, soil, and weather data are available. Logically, this approach can also be applied on other crops by replacing the ORYZA (v3) rice model with the corresponding crop models and following the stepwise process.

Upon integration of the predicted grain yields of all the tested varieties, the environments involved in this study were classified into different drought stress TPEs. In Southern Asia, 86% of the current rice cultivated areas experience mild drought stress, of which 75% occurs at the vegetative stage and only 15% during the whole season if the cropping season was adjusted according to the local soil-climatic condition. Severe drought stress at the reproductive stage could possibly occur in 5.4% of the total area, located mainly in the North Western part (i.e. Pakistan and North West India) of Southern Asia. The TPE classification would be reliable only if the number of tested genotypes was ten or larger. Two GSR varieties, GSR-IR1-1-Y4-Y1 and GSR-IR1-8-S6-S3-Y2 consistently performed better than others across all types of TPE, particularly in TPEs with severe drought stress or drought stress at reproductive stage.

## Supporting Information

S1 Appendix Definition of parameters in S1 Table. (DOCX)

S2 Appendix Checklist of abbreviations used in this paper. (DOCX)

S1 Fig Simulated above-ground biomass (AGB), panicle biomass (PB), and grain yield (GY) against measured values for tested varieties in calibration (A to C) and validation (D to F). (DOCX)

S1 Table Crop parameters generated after minimizing the difference between the simulated and measured crop growth variables by calibration using the Auto-Calibration tool. (DOCX)

S2 Table Statistical results for each variety in comparing the simulated to measured values during calibration. (DOCX)

S3 Table Statistical results for each variety in comparing the simulated to measured values during validation. (DOCX)

S1 Text Validation of model on predicting biomass accumulation and grain yield. (DOCX)
