# Supplementary material for: Combining Limited Multiple Environment Trials Data with Crop Modeling to Identify Widely Adaptable Rice Varieties
Source: PLoS One. 2016 Oct 10;11(10):e0164456. doi: 10.1371/journal.pone.0164456 (PMC5056740; doi:10.1371/journal.pone.0164456)
Supplement: S1 Appendix — (DOCX) [file pone.0164456.s001.docx]

**Appendix S1. Definition of parameters in S1 Table.**

| _DVRJ_ |  | _Development rate in juvenile phase (_^o^_C.d_^-1^_)_ |
| --- | --- | --- |
| _DVRP_ |  | _Development rate in panicle development phase (_^o^_C.d_^-1^_)_ |
| _DVRR_ |  | _Development rate in reproductive phase (_^o^_C.d_^-1^_)_ |
| _RGRLMX_ |  | _Maximum relative growth rate of leaf area (_^o^_C.d_^-1^_)_ |
| _RGRLMN_ |  | _Minimum relative growth rate of leaf area (_^o^_C.d_^-1^_)_ |
| _SHADET_ |  | _Tolerance index to shading (unitless)_ |
| _FSWTD_ |  | _The upper ratio of remaining available water to total water supply capability while transpiration declines (unitless)_ |
| _SLA1 to 7_ |  | _Specific leaf area at different development stages (ha leaf kg_^-1^ _leaf biomass)_ |
| _KDF1 to 4_ |  | _Light extinction coefficient of rice canopy at different development stages (unitless)_ |
| _FST1 to 6_ |  | _Fraction of shoot dry matter allocated to stems at different development stages (unitless)_ |
| _FSO1 to 3_ |  | _Fraction of shoot dry matter allocated to storage organs at different development reproductive stages (unitless)_ |
| _FLV1 to 6_ |  | _Fraction of shoot dry matter allocated to leaves at different development stages (unitless)_ |
| _FSH1, 2_ |  | _Fraction of shoot dry matter allocated to shoots at different vegetative stages (unitless)_ |
| _EFF1 to 4_ |  | _Initial light use efficiency at air temperature 10, 25, 40 and 60_ ^o^_C (unitless)_ |
| _DRLV1 to 4_ |  | _Leaf death coefficient at different reproductive stages (unitless)_ |
