# Supplementary material for: Combining Limited Multiple Environment Trials Data with Crop Modeling to Identify Widely Adaptable Rice Varieties
Source: PLoS One. 2016 Oct 10;11(10):e0164456. doi: 10.1371/journal.pone.0164456 (PMC5056740; doi:10.1371/journal.pone.0164456)
Supplement: S2 Appendix — (DOCX) [file pone.0164456.s002.docx]

**Appendix S2: Checklist of abbreviations used in this paper.**

| **Abbreviation** | **Description** |
| --- | --- |
| *A* | Data matrix of a given variable of simulation outputs for a variety in rainfed condition |
| AGB | Above-ground biomass |
| AR | Data matrix of DIR of simulation outputs for a variety in rainfed condition |
| AR^’^ | Big data matrix of AR for all varieties |
| ar | Data matrix of DIR in the best rainfed season extracted from AR^’^ |
| AV | Data matrix of DIV of simulation outputs for a variety in rainfed condition |
| AV^’^ | Big data matrix of AV for all varieties |
| av | Data matrix of DIV in the best rainfed season extracted from AV^’^ |
| AY | Data matrix of GY of simulation outputs for a variety in rainfed condition |
| AY^’^ | Big data matrix of AY for all varieties |
| ay^’^ | Data matrix of GY in the best rainfed season extracted from AY^’^ |
| C | The experiments for model calibration |
| c | The number of varieties |
| CAAS | Chinese Academy of Agricultural Sciences |
| CF | Continuously flooded |
| CV | Coefficient of variation |
| DIR | Drought stress index in reproductive stage |
| DIV | Drought stress index in vegetative stage |
| DS | Dry season |
| E | The experiments for model evaluation |
| f_75_ | Frequency of rainfed yield occurring less than 75% of irrigated yield |
| GEI | Genotype and environment interaction |
| GSR | Green Super Rice |
| GY | Commercial grain yield (grain biomass at 14% moisture) |
| HHZ | Huang-hua-zhan |
| *i* | Subscript for sowing dates in a year of simulation |
| IRRI | International Rice Research Institute |
| *j* | Subscript for the year of simulation |
| *k* | Subscript for environment in data matrix YP and YA |
| L | None to mild drought stress |
| LAI | Leaf area index |
| LB | Los Baños |
| M | Moderate drought stress |
| m | The maximum number of simulation years |
| M_eff_ | Modeling efficiency |
| MET | Multi-Environment Trials |
| MET_order_ | Varietal performance ranking using MET data |
| n | Maximum number of simulated sowing dates within a calendar year |
| NE | Nueva Ecija |
| *P* | Data matrix of a given variable of simulation outputs under irrigated condition |
| *P(t)* | Student’s t-test |
| *PY* | Data matrix of GY of simulation outputs for a variety in irrigated condition |
| *PY^’^* | Big data matrix of PY for all varieties |
| PB | Panicle biomass |
| P_order_ | Varietal performance rank using simulation data generated from large number of environments |
| q | The total number of environments |
| R | Drought at reproductive stage |
| *R*^2^ | Correlation coefficient |
| RF | Rainfed |
| RMSEn | Root mean square errors between measured and simulated values nominalized by measured mean |
| RY | Rainfed grain yield |
| S | Severe drought stress |
| S_order_ | Varietal performance rank using simulation data generated in MET site-specific condition |
| TPE | Target population environment |
| V | Drought at vegetative stage |
| v | Subscript for variety in simulations |
| V + R | Drought at both vegetative and reproductive stage |
| WISE | World Inventory of Soil Emission Potential |
| WS | Wet season |
| YA | Data matrix of rainfed rice GY of a variety in the best rainfed season |
| YP | Data matrix of irrigated rice GY of a variety in the best rainfed season |
| α | Slope of linear regression |
| β | Intercept of linear regression |
| *γ* | Average rainfed yield derived from YA |
| *δ* | Difference between rainfed rice yield and irrigated rice yield |
| *λ* | Coefficient of variation of rainfed yield in YA |
| *φ* | Spatial variability of yields among environments |
| *ψ* | Temporal variability of yield among different growth seasons |
