# Supplementary material for: Combining Limited Multiple Environment Trials Data with Crop Modeling to Identify Widely Adaptable Rice Varieties
Source: PLoS One. 2016 Oct 10;11(10):e0164456. doi: 10.1371/journal.pone.0164456 (PMC5056740; doi:10.1371/journal.pone.0164456)
Supplement: S1 Fig — (DOCX) [file pone.0164456.s003.docx]

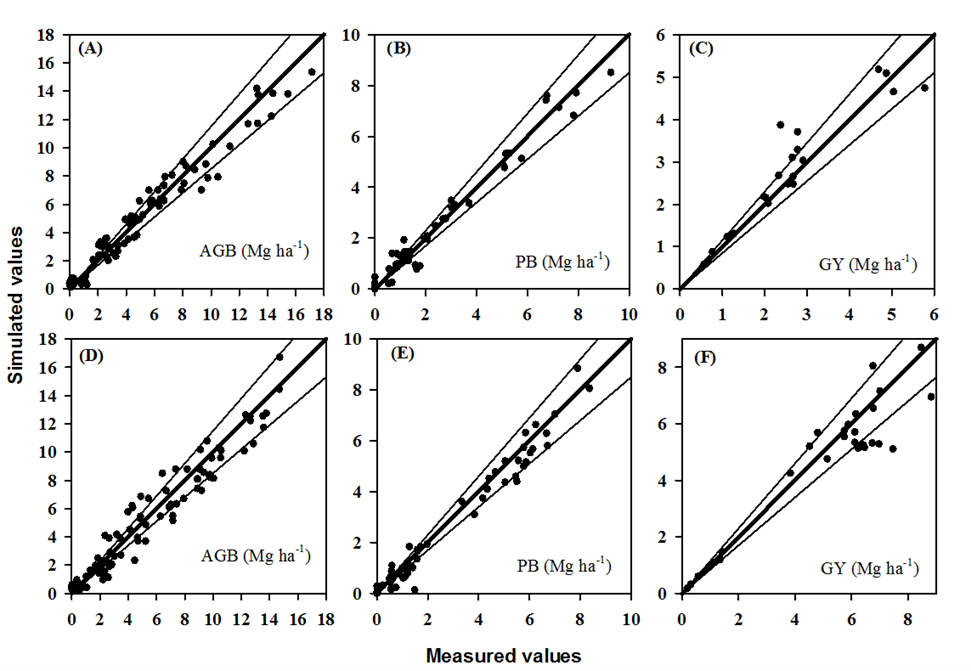


**S1 Fig. Simulated above-ground biomass (AGB), panicle biomass (PB), and grain yield (GY) against measured values for tested varieties in calibration (A to C) and validation (D to F).**
