# Supplementary material for: Combining Limited Multiple Environment Trials Data with Crop Modeling to Identify Widely Adaptable Rice Varieties
Source: PLoS One. 2016 Oct 10;11(10):e0164456. doi: 10.1371/journal.pone.0164456 (PMC5056740; doi:10.1371/journal.pone.0164456)
Supplement: S1 Table — (DOCX) [file pone.0164456.s004.docx]

| **_VARIETY_** | **_FFZ_** | **_GSR-IR1-1-Y4-Y1_** | **_GSR-IR1-5-S8-D3-SUB1_** | **_GSR-IR1-5-S10-D1-D1_** | **_GSR-IR1-5-S14-S2-Y2_** | **_GSR-IR1-8-S6-S3-Y2_** | **_GSR-IR1-8-S12-Y2-D1_** | **_GSR-IR1-12-D10-S1-D1_** | **_IR-74371-70-1-1_** | **_NSICRC158_** | **_PSBRC82_** |
| --- | --- | --- | --- | --- | --- | --- | --- | --- | --- | --- | --- |
| _DVRJ_ | _0.000576_ | _0.000592_ | _0.000643_ | _0.000592_ | _0.000592_ | _0.000685_ | _0.000625_ | _0.000561_ | _0.000576_ | _0.000519_ | _0.00058_ |
| _DVRP_ | _0.00075_ | _0.00075_ | _0.000754_ | _0.00075_ | _0.00075_ | _0.000819_ | _0.000784_ | _0.00075_ | _0.00075_ | _0.000944_ | _0.000646_ |
| _DVRR_ | _0.002037_ | _0.00184_ | _0.001828_ | _0.002036_ | _0.001963_ | _0.001888_ | _0.001769_ | _0.002116_ | _0.001787_ | _0.001932_ | _0.003255_ |
| _RGRLMX_ | _0.005558_ | _0.008613_ | _0.004303_ | _0.009424_ | _0.009195_ | _0.009391_ | _0.00626_ | _0.01137_ | _0.005388_ | _0.010973_ | _0.009907_ |
| _RGRLMN_ | _0.006367_ | _0.004404_ | _0.004841_ | _0.003761_ | _0.005482_ | _0.005849_ | _0.002698_ | _0.003551_ | _0.004299_ | _0.003606_ | _0.003064_ |
| _SHADET_ | _0.888554_ | _0.11076_ | _0.40972_ | _0.983685_ | _0.578396_ | _0.397914_ | _0.480978_ | _0.303176_ | _0.480978_ | _0.945548_ | _0.846498_ |
| _FSWTD_ | _0.583522_ | _0.205962_ | _0.444286_ | _0.575547_ | _0.496572_ | _0.554613_ | _0.583522_ | _0.575547_ | _0.583522_ | _0.526142_ | _0.461255_ |
| _SLA1_ | _0.006511_ | _0.006351_ | _0.002891_ | _0.006732_ | _0.003_ | _0.005263_ | _0.003_ | _0.004272_ | _0.005776_ | _0.006511_ | _0.006511_ |
| _SLA2_ | _0.002586_ | _0.00523_ | _0.00426_ | _0.004299_ | _0.002586_ | _0.004457_ | _0.005381_ | _0.006648_ | _0.004751_ | _0.00386_ | _0.005915_ |
| _SLA3_ | _0.001962_ | _0.001908_ | _0.004412_ | _0.002707_ | _0.002032_ | _0.003232_ | _0.002191_ | _0.003592_ | _0.001811_ | _0.003031_ | _0.001854_ |
| _SLA4_ | _0.003628_ | _0.002873_ | _0.001636_ | _0.003485_ | _0.004178_ | _0.001531_ | _0.002552_ | _0.001864_ | _0.003097_ | _0.003314_ | _0.003064_ |
| _SLA5_ | _0.001766_ | _0.001799_ | _0.002929_ | _0.0017_ | _0.001238_ | _0.002989_ | _0.001716_ | _0.001976_ | _0.002989_ | _0.003254_ | _0.002949_ |
| _SLA6_ | _0.001405_ | _0.001198_ | _0.001956_ | _0.00142_ | _0.001405_ | _0.00125_ | _0.001341_ | _0.001224_ | _0.00125_ | _0.001451_ | _0.001224_ |
| _SLA7_ | _0.002017_ | _0.002949_ | _0.002368_ | _0.003281_ | _0.002017_ | _0.001654_ | _0.001769_ | _0.002846_ | _0.001654_ | _0.002636_ | _0.003032_ |
| _KDF1_ | _0.556076_ | _0.418164_ | _0.328647_ | _0.475288_ | _0.55654_ | _0.335665_ | _0.453673_ | _0.586622_ | _0.574781_ | _0.487883_ | _0.320165_ |
| _KDF2_ | _0.477862_ | _0.447165_ | _0.340788_ | _0.368907_ | _0.466879_ | _0.367225_ | _0.466047_ | _0.586761_ | _0.405611_ | _0.477642_ | _0.480609_ |
| _KDF3_ | _0.48249_ | _0.514166_ | _0.446328_ | _0.542726_ | _0.71427_ | _0.577072_ | _0.413166_ | _0.423252_ | _0.471001_ | _0.745361_ | _0.691388_ |
| _KDF4_ | _0.629478_ | _0.622271_ | _0.595391_ | _0.413851_ | _0.66201_ | _0.624085_ | _0.487471_ | _0.677095_ | _0.508263_ | _0.588591_ | _0.536421_ |
| _FST1_ | _0.637402_ | _0.468402_ | _0.309064_ | _0.627245_ | _0.086708_ | _0.949695_ | _0.532459_ | _0.82828_ | _0.578025_ | _0.537756_ | _0.384795_ |
| _FST2_ | _0.369095_ | _0.380328_ | _0.700328_ | _0.442178_ | _0.627384_ | _0.303207_ | _0.584981_ | _0.301029_ | _0.348067_ | _0.590965_ | _0.567488_ |
| _FST3_ | _0.826252_ | _0.757998_ | _0.567994_ | _0.565683_ | _0.316128_ | _0.764022_ | _0.481777_ | _0.497864_ | _0.83299_ | _0.692034_ | _0.571419_ |
| _FST4_ | _0.148566_ | _0.419439_ | _0.723044_ | _0.246164_ | _0.517172_ | _0.225341_ | _0.731899_ | _0.621256_ | _0.334103_ | _0.450279_ | _0.502952_ |
| _FST5_ | _0.173703_ | _0.037671_ | _0.145826_ | _0.278918_ | _0.271151_ | _0.154785_ | _0.438159_ | _0.195977_ | _0.215582_ | _0.0000_ | _0.0000_ |
| _FST6_ | _0.025366_ | _0.062068_ | _0.013106_ | _0.025004_ | _0.264107_ | _0.123236_ | _0.15701_ | _0.254507_ | _0.145909_ | _0.0000_ | _0.0000_ |
| _FSO1_ | _0.499304_ | _0.505279_ | _0.19518_ | _0.710925_ | _0.477083_ | _0.530185_ | _0.261987_ | _0.371806_ | _0.378298_ | _0.54676_ | _0.474554_ |
| _FSO2_ | _0.815421_ | _0.936741_ | _0.853821_ | _0.707753_ | _0.721763_ | _0.837126_ | _0.556229_ | _0.758782_ | _0.781629_ | _0.984034_ | _0.99783_ |
| _FSO3_ | _0.87832_ | _0.932692_ | _0.961273_ | _0.965514_ | _0.73104_ | _0.876041_ | _0.838916_ | _0.733065_ | _0.851806_ | _0.958194_ | _0.976049_ |
| _FLV1_ | _0.362598_ | _0.531598_ | _0.690936_ | _0.372755_ | _0.913292_ | _0.050305_ | _0.467541_ | _0.17172_ | _0.421975_ | _0.462244_ | _0.615205_ |
| _FLV2_ | _0.630905_ | _0.619672_ | _0.299672_ | _0.557823_ | _0.372617_ | _0.696793_ | _0.415019_ | _0.698971_ | _0.651933_ | _0.409035_ | _0.432512_ |
| _FLV3_ | _0.173748_ | _0.242002_ | _0.432006_ | _0.434317_ | _0.683872_ | _0.235978_ | _0.518223_ | _0.502136_ | _0.16701_ | _0.307966_ | _0.428581_ |
| _FLV4_ | _0.352131_ | _0.075282_ | _0.081777_ | _0.042911_ | _0.005745_ | _0.244474_ | _0.006114_ | _0.006938_ | _0.287599_ | _0.002961_ | _0.022494_ |
| _FLV5_ | _0.010876_ | _0.025588_ | _0.000352_ | _0.013329_ | _0.007087_ | _0.00809_ | _0.005613_ | _0.045242_ | _0.00279_ | _0.015966_ | _0.00217_ |
| _FLV6_ | _0.096315_ | _0.00524_ | _0.02562_ | _0.009482_ | _0.004853_ | _0.000723_ | _0.004075_ | _0.012428_ | _0.002286_ | _0.041806_ | _0.023951_ |
| _FSH1_ | _0.707455_ | _0.364893_ | _0.691532_ | _0.574294_ | _0.428393_ | _0.377684_ | _0.592151_ | _0.273162_ | _0.806764_ | _0.259346_ | _0.256797_ |
| _FSH2_ | _0.257866_ | _0.560017_ | _0.861535_ | _0.390906_ | _0.24009_ | _0.456377_ | _0.376054_ | _0.305524_ | _0.556109_ | _0.600221_ | _0.467058_ |
| _EFF1_ | _0.456199_ | _0.665733_ | _0.450605_ | _0.385896_ | _0.512803_ | _0.748335_ | _0.502791_ | _0.546037_ | _0.627907_ | _0.52038_ | _0.600352_ |
| _EFF2_ | _0.797851_ | _0.392899_ | _0.556346_ | _0.55659_ | _0.490475_ | _0.645618_ | _0.596037_ | _0.688772_ | _0.537061_ | _0.643368_ | _0.570274_ |
| _EFF3_ | _0.432504_ | _0.328128_ | _0.277579_ | _0.396906_ | _0.444718_ | _0.397385_ | _0.537607_ | _0.430825_ | _0.381626_ | _0.430437_ | _0.418202_ |
| _EFF4_ | _0.22931_ | _0.340332_ | _0.268136_ | _0.207755_ | _0.220976_ | _0.324837_ | _0.197347_ | _0.225425_ | _0.156439_ | _0.275962_ | _0.21562_ |
| _DRLV1_ | _0.016523_ | _0.020227_ | _0.015168_ | _0.016683_ | _0.026411_ | _0.02772_ | _0.012223_ | _0.007864_ | _0.017132_ | _0.017876_ | _0.013079_ |
| _DRLV2_ | _0.036642_ | _0.023506_ | _0.024208_ | _0.031905_ | _0.038909_ | _0.011325_ | _0.00838_ | _0.033481_ | _0.021525_ | _0.022471_ | _0.019685_ |
| _DRLV3_ | _0.107422_ | _0.092395_ | _0.03485_ | _0.071281_ | _0.075079_ | _0.067897_ | _0.037518_ | _0.04957_ | _0.053786_ | _0.026005_ | _0.074793_ |
| _DRLV4_ | _0.051473_ | _0.043921_ | _0.03064_ | _0.041392_ | _0.064798_ | _0.072548_ | _0.085154_ | _0.050217_ | _0.057999_ | _0.073341_ | _0.054484_ |

**S1 Table.** The crop parameters generated after minimizing the difference between the simulated and measured crop growth variables by calibration using the Auto-Calibration tool. The crop growth variables, AGB, WST, WLVG, WLVD and PB are the biomass of total above-ground plant, stem, green and dead leaves, panicles, while LAI is the leaf area index.
