# Supplementary material for: Combining Limited Multiple Environment Trials Data with Crop Modeling to Identify Widely Adaptable Rice Varieties
Source: PLoS One. 2016 Oct 10;11(10):e0164456. doi: 10.1371/journal.pone.0164456 (PMC5056740; doi:10.1371/journal.pone.0164456)
Supplement: S2 Table — (DOCX) [file pone.0164456.s005.docx]

| **Variety** | **Crop growth variable** | **n** | **R^2^** | **α** | **β** | | **p(t)** | | **RMSEn** | | **Meff** | |  |
| --- | --- | --- | --- | --- | --- | --- | --- | --- | --- | --- | --- | --- | --- |
| FFZ | AGB | 10 | 0.948 | 402.328 | | 0.858 | | 0.463 | | 16.784 | | 0.994 | |
|  | PB | 4 | 0.950 | 204.964 | | 0.945 | | 0.496 | | 4.576 | | 0.999 | |
|  | WST | 10 | 0.913 | 139.769 | | 0.856 | | 0.440 | | 15.956 | | 0.993 | |
|  | WLVD | 6 | 0.794 | -43.478 | | 1.223 | | 0.438 | | 21.306 | | 0.987 | |
|  | WLVG | 10 | 0.815 | 274.573 | | 0.534 | | 0.375 | | 54.057 | | 0.902 | |
|  | LAI | 10 | 0.849 | 0.604 | | 0.518 | | 0.392 | | 60.756 | | 0.895 | |
| HHZ_1-Y4-Y1 | AGB | 5 | 0.971 | 64.413 | | 1.033 | | 0.462 | | 6.021 | | 0.999 | |
|  | PB | 3 | 1.000 | 104.851 | | 0.992 | | 0.464 | | 8.923 | | 0.998 | |
|  | WST | 5 | 0.842 | 52.046 | | 0.923 | | 0.468 | | 6.860 | | 0.997 | |
|  | WLVD | 3 | 0.338 | -948.428 | | 3.018 | | 0.447 | | 29.170 | | 0.733 | |
|  | WLVG | 5 | 0.901 | 137.114 | | 1.007 | | 0.309 | | 29.255 | | 0.966 | |
|  | LAI | 5 | 0.601 | 0.737 | | 0.511 | | 0.286 | | 59.046 | | 0.851 | |
| HHZ_5-SAL8-DT3-SUB1 | AGB | 5 | 0.967 | -334.263 | | 1.023 | | 0.439 | | 8.792 | | 0.997 | |
|  | PB | 3 | 0.966 | 328.240 | | 0.842 | | 0.478 | | 17.405 | | 0.992 | |
|  | WST | 5 | 0.900 | -171.726 | | 0.960 | | 0.358 | | 16.654 | | 0.981 | |
|  | WLVD | 3 | 0.253 | 1409.763 | | -2.126 | | 0.159 | | 42.052 | | 0.068 | |
|  | WLVG | 5 | 0.810 | 120.655 | | 0.762 | | 0.485 | | 19.497 | | 0.982 | |
|  | LAI | 5 | 0.895 | 0.378 | | 0.749 | | 0.412 | | 26.628 | | 0.973 | |
| HHZ_5-SAL10-DT1-DT1 | AGB | 10 | 0.974 | 120.481 | | 1.068 | | 0.409 | | 12.644 | | 0.996 | |
|  | PB | 4 | 0.998 | 201.695 | | 0.893 | | 0.488 | | 8.331 | | 0.997 | |
|  | WST | 11 | 0.858 | 139.913 | | 1.046 | | 0.352 | | 15.698 | | 0.992 | |
|  | WLVD | 5 | 0.449 | 223.534 | | 0.621 | | 0.473 | | 32.352 | | 0.944 | |
|  | WLVG | 10 | 0.826 | 204.650 | | 1.031 | | 0.185 | | 45.320 | | 0.950 | |
|  | LAI | 10 | 0.916 | 0.618 | | 0.868 | | 0.225 | | 42.778 | | 0.962 | |
| HHZ_5_SAL14-SAL2-Y2 | AGB | 5 | 0.961 | 75.010 | | 1.089 | | 0.427 | | 13.798 | | 0.994 | |
|  | PB | 3 | 1.000 | 176.010 | | 0.927 | | 0.471 | | 10.159 | | 0.997 | |
|  | WST | 5 | 0.891 | -32.593 | | 1.015 | | 0.492 | | 1.610 | | 1.000 | |
|  | WLVD | 2 | 1.000 | -1917.747 | | 5.653 | | 0.393 | | 80.507 | | 0.469 | |
|  | WLVG | 5 | 0.778 | 69.017 | | 1.339 | | 0.251 | | 56.330 | | 0.910 | |
|  | LAI | 5 | 0.689 | 0.601 | | 0.737 | | 0.233 | | 54.696 | | 0.901 | |
| HHZ_8-SAL6-SAL3-Y2 | AGB | 5 | 0.989 | 90.912 | | 1.031 | | 0.464 | | 6.502 | | 0.999 | |
|  | PB | 3 | 0.998 | 58.112 | | 1.042 | | 0.466 | | 8.974 | | 0.998 | |
|  | WST | 5 | 0.960 | 179.391 | | 0.844 | | 0.470 | | 14.153 | | 0.992 | |
|  | WLVD | 3 | 0.663 | -657.311 | | 2.820 | | 0.271 | | 48.125 | | 0.631 | |
|  | WLVG | 5 | 0.982 | 141.970 | | 0.906 | | 0.347 | | 21.764 | | 0.977 | |
|  | LAI | 5 | 0.971 | 0.637 | | 0.788 | | 0.274 | | 50.609 | | 0.939 | |
| HHZ_8-SAL12-Y2-DT1 | AGB | 10 | 0.980 | 222.351 | | 0.925 | | 0.476 | | 7.562 | | 0.998 | |
|  | PB | 5 | 0.986 | 198.838 | | 0.918 | | 0.484 | | 8.063 | | 0.998 | |
|  | WST | 10 | 0.968 | 55.614 | | 0.922 | | 0.446 | | 8.870 | | 0.997 | |
|  | WLVD | 6 | 0.712 | 51.318 | | 0.903 | | 0.486 | | 7.037 | | 0.997 | |
|  | WLVG | 10 | 0.937 | 251.006 | | 0.679 | | 0.434 | | 32.338 | | 0.962 | |
|  | LAI | 10 | 0.964 | 0.552 | | 0.729 | | 0.469 | | 29.516 | | 0.975 | |
| HHZ_12-DT10-SAL1-DT1 | AGB | 10 | 0.979 | 238.767 | | 0.967 | | 0.481 | | 4.085 | | 1.000 | |
|  | PB | 5 | 0.999 | 219.977 | | 0.959 | | 0.475 | | 6.153 | | 0.999 | |
|  | WST | 10 | 0.893 | 177.271 | | 0.926 | | 0.481 | | 6.992 | | 0.998 | |
|  | WLVD | 6 | 0.401 | 125.542 | | 0.759 | | 0.429 | | 18.980 | | 0.976 | |
|  | WLVG | 10 | 0.957 | 193.119 | | 0.901 | | 0.402 | | 14.447 | | 0.994 | |
|  | LAI | 10 | 0.915 | 0.831 | | 0.707 | | 0.379 | | 34.071 | | 0.965 | |
| IR_74371-70-1-1 | AGB | 10 | 0.977 | 202.591 | | 0.915 | | 0.471 | | 8.637 | | 0.998 | |
|  | PB | 5 | 0.995 | 230.136 | | 0.866 | | 0.482 | | 12.696 | | 0.995 | |
|  | WST | 10 | 0.939 | 96.575 | | 0.890 | | 0.444 | | 10.685 | | 0.995 | |
|  | WLVD | 5 | 0.657 | 74.704 | | 1.120 | | 0.317 | | 37.079 | | 0.958 | |
|  | WLVG | 10 | 0.822 | 169.293 | | 0.783 | | 0.499 | | 18.964 | | 0.985 | |
|  | LAI | 10 | 0.875 | 0.379 | | 0.834 | | 0.462 | | 16.584 | | 0.991 | |
| NSICRc158 | AGB | 16 | 0.979 | 423.712 | | 0.892 | | 0.474 | | 12.109 | | 0.997 | |
|  | PB | 7 | 0.974 | -185.979 | | 1.018 | | 0.475 | | 3.763 | | 1.000 | |
|  | WST | 16 | 0.969 | 259.691 | | 0.856 | | 0.465 | | 13.700 | | 0.994 | |
|  | WLVD | 9 | 0.359 | 309.993 | | 0.328 | | 0.451 | | 105.191 | | 0.743 | |
|  | WLVG | 16 | 0.914 | 289.095 | | 0.661 | | 0.461 | | 31.165 | | 0.958 | |
|  | LAI | 15 | 0.803 | 1.121 | | 0.796 | | 0.157 | | 43.329 | | 0.948 | |
| PSBRc82 | AGB | 16 | 0.971 | 148.122 | | 0.911 | | 0.437 | | 10.528 | | 0.997 | |
|  | PB | 8 | 0.953 | 123.020 | | 0.952 | | 0.492 | | 4.271 | | 0.999 | |
|  | WST | 15 | 0.938 | 163.668 | | 0.813 | | 0.354 | | 18.764 | | 0.984 | |
|  | WLVD | 10 | 0.766 | 148.206 | | 0.769 | | 0.377 | | 28.701 | | 0.977 | |
|  | WLVG | 16 | 0.937 | 209.386 | | 0.646 | | 0.371 | | 35.965 | | 0.950 | |
|  | LAI | 15 | 0.863 | 0.660 | | 0.658 | | 0.446 | | 37.003 | | 0.957 | |

**S2 Table.** Statistical results for each variety in comparing the simulated to measured values during calibration. AGB, PB, WST, WLVG, and WLVD are the crop growth variables on biomass of above-ground plant, panicles, stem, and green and dead leaves. LAI is the leaf area index. The *n*, α*,* $\beta$, *p(t)*, *RMSE_n_* , and *Meff* are the statistical indicators for data pairs, correlation coefficient, the intercept and slope of linear regression, student-t test with unequal mean assumption, root mean square error normalized by measured mean, and modeling efficiency, respectively.
