# Supplementary material for: Combining Limited Multiple Environment Trials Data with Crop Modeling to Identify Widely Adaptable Rice Varieties
Source: PLoS One. 2016 Oct 10;11(10):e0164456. doi: 10.1371/journal.pone.0164456 (PMC5056740; doi:10.1371/journal.pone.0164456)
Supplement: S3 Table — (DOCX) [file pone.0164456.s006.docx]

| **Variety** | **Crop growth variable** | **n** | **R^2^** | **α** | **β** | **p(t)** | **RMSE_n_** | **Meff** |
| --- | --- | --- | --- | --- | --- | --- | --- | --- |
| FFZ | AGB | 10 | 0.875 | 184.422 | 1.057 | 0.389 | 13.808 | 0.994 |
|  | PB | 4 | 0.993 | -161.013 | 0.981 | 0.443 | 12.108 | 0.996 |
|  | WST | 10 | 0.869 | 48.925 | 1.165 | 0.334 | 25.354 | 0.983 |
|  | WLVD | 4 | 0.373 | 160.257 | 0.456 | 0.256 | 46.139 | 0.768 |
|  | WLVG | 10 | 0.564 | 329.610 | 0.837 | 0.143 | 55.491 | 0.911 |
|  | LAI | 10 | 0.438 | 0.928 | 0.559 | 0.204 | 69.663 | 0.872 |
| HHZ_1-Y4-Y1 | AGB | 5 | 0.998 | 160.670 | 0.869 | 0.447 | 15.155 | 0.993 |
|  | PB | 2 | 1.000 | -345.064 | 0.926 | 0.435 | 18.274 | 0.982 |
|  | WST | 5 | 0.979 | -6.765 | 0.955 | 0.468 | 6.377 | 0.999 |
|  | WLVD | 2 | 1.000 | -377.053 | 0.958 | 0.254 | 37.170 | 0.771 |
|  | WLVG | 5 | 0.905 | 273.308 | 0.775 | 0.334 | 36.324 | 0.959 |
|  | LAI | 5 | 0.935 | 0.858 | 0.454 | 0.458 | 59.450 | 0.858 |
| HHZ_5-SAL8-DT3-SUB1 | AGB | 5 | 0.997 | -72.609 | 0.918 | 0.441 | 13.287 | 0.995 |
|  | PB | 3 | 0.994 | -229.499 | 0.957 | 0.454 | 15.109 | 0.996 |
|  | WST | 5 | 0.960 | -79.129 | 1.077 | 0.495 | 6.444 | 0.999 |
|  | WLVD | 2 | 1.000 | -469.029 | 0.804 | 0.131 | 59.365 | 0.579 |
|  | WLVG | 5 | 0.834 | 176.711 | 0.555 | 0.423 | 46.753 | 0.913 |
|  | LAI | 5 | 0.950 | 0.387 | 0.841 | 0.431 | 20.913 | 0.988 |
| HHZ_5-SAL10-DT1-DT1 | AGB | 10 | 0.917 | 295.666 | 0.919 | 0.490 | 8.289 | 0.998 |
|  | PB | 5 | 0.999 | 274.654 | 0.968 | 0.457 | 8.263 | 0.998 |
|  | WST | 11 | 0.772 | 277.906 | 0.898 | 0.451 | 9.905 | 0.996 |
|  | WLVD | 5 | 0.646 | 48.265 | 1.029 | 0.415 | 15.125 | 0.992 |
|  | WLVG | 10 | 0.729 | 296.255 | 0.467 | 0.314 | 62.171 | 0.854 |
|  | LAI | 10 | 0.920 | 0.543 | 0.812 | 0.408 | 25.601 | 0.986 |
| HHZ_5_SAL14-SAL2-Y2 | AGB | 5 | 0.986 | -153.141 | 0.877 | 0.408 | 20.217 | 0.988 |
|  | PB | 2 | 1.000 | -159.462 | 0.942 | 0.461 | 10.949 | 0.993 |
|  | WST | 5 | 0.979 | -23.984 | 0.719 | 0.298 | 38.421 | 0.936 |
|  | WLVD | 2 | 1.000 | -391.160 | 1.045 | 0.360 | 22.533 | 0.922 |
|  | WLVG | 5 | 0.938 | 170.299 | 0.805 | 0.455 | 21.762 | 0.987 |
|  | LAI | 5 | 1.000 | 0.582 | 0.821 | 0.359 | 31.681 | 0.973 |
| HHZ_8-SAL6-SAL3-Y2 | AGB | 5 | 0.960 | 129.142 | 0.864 | 0.435 | 16.423 | 0.991 |
|  | PB | 3 | 0.995 | -121.838 | 0.949 | 0.461 | 11.593 | 0.997 |
|  | WST | 5 | 0.956 | 250.142 | 0.810 | 0.479 | 15.425 | 0.989 |
|  | WLVD | 2 | 1.000 | -445.104 | 0.835 | 0.196 | 49.586 | 0.688 |
|  | WLVG | 5 | 0.790 | 318.268 | 0.524 | 0.486 | 47.909 | 0.902 |
|  | LAI | 5 | 0.789 | 0.987 | 0.539 | 0.416 | 52.325 | 0.904 |
| HHZ_8-SAL12-Y2-DT1 | AGB | 10 | 0.932 | 300.471 | 1.009 | 0.422 | 9.614 | 0.998 |
|  | PB | 4 | 0.990 | 310.488 | 0.941 | 0.459 | 9.203 | 0.997 |
|  | WST | 10 | 0.859 | 142.849 | 0.910 | 0.496 | 8.620 | 0.998 |
|  | WLVD | 4 | 0.328 | 477.622 | 0.254 | 0.275 | 59.389 | 0.584 |
|  | WLVG | 10 | 0.687 | 462.925 | 0.862 | 0.097 | 70.287 | 0.888 |
|  | LAI | 10 | 0.760 | 0.948 | 0.674 | 0.188 | 63.169 | 0.915 |
| HHZ_12-DT10-SAL1-DT1 | AGB | 10 | 0.974 | 256.200 | 0.982 | 0.439 | 7.617 | 0.999 |
|  | PB | 4 | 0.983 | -143.226 | 1.042 | 0.480 | 5.554 | 0.999 |
|  | WST | 10 | 0.949 | 20.690 | 1.055 | 0.436 | 8.638 | 0.998 |
|  | WLVD | 6 | 0.686 | 82.533 | 0.831 | 0.480 | 14.122 | 0.991 |
|  | WLVG | 10 | 0.868 | 267.221 | 0.853 | 0.203 | 41.801 | 0.953 |
|  | LAI | 10 | 0.837 | 0.914 | 0.579 | 0.220 | 61.901 | 0.894 |
| IR_74371-70-1-1 | AGB | 10 | 0.979 | 165.510 | 1.021 | 0.438 | 8.220 | 0.998 |
|  | PB | 5 | 0.990 | -16.647 | 0.886 | 0.437 | 17.832 | 0.993 |
|  | WST | 10 | 0.859 | 110.301 | 1.185 | 0.282 | 32.616 | 0.972 |
|  | WLVD | 6 | 0.491 | 216.841 | 0.462 | 0.340 | 51.495 | 0.845 |
|  | WLVG | 10 | 0.738 | 305.679 | 0.648 | 0.266 | 49.633 | 0.930 |
|  | LAI | 10 | 0.913 | 0.613 | 0.775 | 0.328 | 34.835 | 0.972 |
| NSICRc158 | AGB | 18 | 0.971 | -68.584 | 0.966 | 0.442 | 5.503 | 0.999 |
|  | PB | 9 | 0.954 | -329.908 | 1.062 | 0.479 | 3.974 | 0.999 |
|  | WST | 18 | 0.970 | 55.865 | 0.886 | 0.369 | 13.375 | 0.993 |
|  | WLVD | 10 | 0.352 | 655.876 | 0.408 | 0.150 | 62.875 | 0.769 |
|  | WLVG | 18 | 0.436 | 480.576 | 0.426 | 0.361 | 54.435 | 0.834 |
|  | LAI | 18 | 0.701 | 1.325 | 0.608 | 0.215 | 49.208 | 0.920 |
| PSBRc82 | AGB | 28 | 0.962 | 168.374 | 0.900 | 0.403 | 11.978 | 0.996 |
|  | PB | 14 | 0.967 | -219.398 | 0.961 | 0.367 | 10.760 | 0.995 |
|  | WST | 28 | 0.922 | 211.007 | 0.826 | 0.388 | 18.140 | 0.989 |
|  | WLVD | 14 | 0.570 | 207.344 | 0.653 | 0.445 | 27.735 | 0.955 |
|  | WLVG | 28 | 0.509 | 388.411 | 0.541 | 0.369 | 44.532 | 0.909 |
|  | LAI | 28 | 0.600 | 1.072 | 0.547 | 0.193 | 54.634 | 0.898 |

**S3 Table.** Statistical results for each variety in comparing the simulated to measured values during validation. AGB, PB, WST, WLVG, and WLVD are the crop growth variables on biomass of above-ground plant, panicles, stem, and green and dead leaves. LAI is the leaf area index. The *n*, α*,* β, *p(t)*, *RMSE_n_* , and *Meff* are the statistical indicators for data pairs, correlation coefficient, the intercept and slope of linear regression, student-t test with unequal mean assumption, root mean square error normalized by measured mean, and modeling efficiency, respectively.
