# Supplementary material for: Combining Limited Multiple Environment Trials Data with Crop Modeling to Identify Widely Adaptable Rice Varieties
Source: PLoS One. 2016 Oct 10;11(10):e0164456. doi: 10.1371/journal.pone.0164456 (PMC5056740; doi:10.1371/journal.pone.0164456)
Supplement: S1 Text — (DOCX) [file pone.0164456.s007.docx]

## S1 Text. Validation of model on predicting biomass accumulation and grain yield

The measured and simulated AGB, PB, and GY for each variety had good 1:1 relationship, and more than 90% of them were in the range of 15% uncertainty of measured values for both calibration (S1A-S1C Figs) and validation (S1D-S1F Figs) datasets. Scatter plots in S1C and S1F Figs showed that the simulations on GY have uncertainties of up to 20% of the measured values for all varieties.
